# Rapid birth-and-death evolution of the xenobiotic metabolizing *NAT* gene family in vertebrates with evidence of adaptive selection

**DOI:** 10.1186/1471-2148-13-62

**Published:** 2013-03-07

**Authors:** Audrey Sabbagh, Julie Marin, Charlotte Veyssière, Emilie Lecompte, Sotiria Boukouvala, Estella S Poloni, Pierre Darlu, Brigitte Crouau-Roy

**Affiliations:** 1 Institut de Recherche pour le Développement (IRD), UMR216-Mère et enfant face aux infections tropicales, Paris, France; 2 PRES Paris Sorbonne Cité, Université Paris Descartes, Paris, France; 3 CNRS, Université Paul Sabatier, ENFA, UMR5174EDB (Laboratoire Évolution & Diversité Biologique), Toulouse F-31062, France; 4 Université de Toulouse 3, UMR5174EDB, Toulouse F-31062, France; 5 Department of Molecular Biology and Genetics, Democritus University of Thrace, Alexandroupolis, Greece; 6 Laboratory of Anthropology, Genetics and Peopling History, Anthropology Unit, Department of Genetics and Evolution, University of Geneva, Geneva, Switzerland; 7 UMR 7206 Eco-anthropologie et ethnobiologie, MNHN-CNRS-Université Denis Diderot, Paris, France; 8 IRD UMR 216, Université Paris Descartes, Paris, France

**Keywords:** *N*-acetyltransferases, Gene family, Vertebrates, Gene duplication, Adaptive selection

## Abstract

**Background:**

The arylamine N-acetyltransferases (NATs) are a unique family of enzymes widely distributed in nature that play a crucial role in the detoxification of aromatic amine xenobiotics. Considering the temporal changes in the levels and toxicity of environmentally available chemicals, the metabolic function of NATs is likely to be under adaptive evolution to broaden or change substrate specificity over time, making NATs a promising subject for evolutionary analyses. In this study, we trace the molecular evolutionary history of the *NAT* gene family during the last ~450 million years of vertebrate evolution and define the likely role of gene duplication, gene conversion and positive selection in the evolutionary dynamics of this family.

**Results:**

A phylogenetic analysis of 77 *NAT* sequences from 38 vertebrate species retrieved from public genomic databases shows that *NAT*s are phylogenetically unstable genes, characterized by frequent gene duplications and losses even among closely related species, and that concerted evolution only played a minor role in the patterns of sequence divergence. Local signals of positive selection are detected in several lineages, probably reflecting response to changes in xenobiotic exposure. We then put a special emphasis on the study of the last ~85 million years of primate *NAT* evolution by determining the *NAT* homologous sequences in 13 additional primate species. Our phylogenetic analysis supports the view that the three human *NAT* genes emerged from a first duplication event in the common ancestor of Simiiformes, yielding *NAT1* and an ancestral *NAT* gene which in turn, duplicated in the common ancestor of Catarrhini, giving rise to *NAT2* and the *NATP* pseudogene. Our analysis suggests a main role of purifying selection in NAT1 protein evolution, whereas NAT2 was predicted to mostly evolve under positive selection to change its amino acid sequence over time. These findings are consistent with a differential role of the two human isoenzymes and support the involvement of NAT1 in endogenous metabolic pathways.

**Conclusions:**

This study provides unequivocal evidence that the *NAT* gene family has evolved under a dynamic process of birth-and-death evolution in vertebrates, consistent with previous observations made in fungi.

## Background

Arylamine *N*-acetyltransferases (NATs) are xenobiotic metabolizing enzymes found in a wide range of species across all major clades of life (bacteria, archaea, eukaryotes), except plants [1-3]. This unique family of enzymes catalyses the transfer of an acetyl group from acetyl-coenzyme A (acetyl-CoA) to the terminal nitrogen of arylamine, hydrazine and heterocyclic amine compounds. Biochemical, structural and functional studies of the last decades have increased our understanding of the potentially diverse roles of NATs in endogenous and xenobiotic metabolism, establishing their key role in cellular homeostasis as well as in gene-environment interactions [4-6].

Humans have two functional *NAT* genes (*NAT1* and *NAT2*) and one pseudogene (*NATP*), located within 200 kilobases (kb) on chromosome 8 [7,8]. Both *NAT1* and *NAT2* genes have intronless coding regions with a single coding exon of 870 base pairs (bp) that produces a 290 amino acid (34 kDa) protein. They share 81% nucleotide identity, which translates to 87% identity at the amino acid level. The pseudogene *NATP* displays high sequence identity to *NAT1* and *NAT2* (79%) but contains multiple frameshift and premature stop codon mutations leading to loss of function [9]. Despite their high degree of sequence identity, *NAT1* and *NAT2* encode isoenzymes with distinct substrate specificities, tissue distribution and expression levels during development. NAT1 is widely expressed in most tissues and cell types and preferentially acetylates substrates such as *p*-aminobenzoic acid (pABA), *p*-aminosalicylic acid (pASA) and *p*-aminobenzoylglutamate (pABG). NAT2 has a more restricted expression profile, being predominantly expressed in the liver, small intestine and colon, and prefers bulkier substrates such as sulphamethazine, isoniazid, procainamide, and dapsone (reviewed in [10]). The widespread tissue distribution of NAT1, its selectivity for pABG as well as its early expression in development (as early as the blastocyst stage [11]), have suggested that NAT1 may have an endogenous role in addition to the metabolism of xenobiotics. Acetylating the folate breakdown product pABG is now generally accepted to be an endogenous role of this enzyme which might be important in normal embryonic development [12,13]. The importance of NAT1 and NAT2 in the metabolism of drugs and in the activation of common environmental carcinogens has led to a plethora of molecular epidemiological studies that have shown associations of *NAT* gene polymorphisms with individual drug response and susceptibility to cancers linked to arylamine exposure [14-16].

Because of their role in the detoxification of exogenous chemicals present in the diet and the environment, human *NAT1* and *NAT2* genes have long been considered as likely targets of population-specific selective pressures, a hypothesis further fuelled by the intriguing patterns of geographic differentiation of their major alleles [17-24]. In particular, the *NAT2* gene, which exhibits a well established acetylation polymorphism leading to a phenotype classification of individuals into rapid and slow acetylators, has been the subject of numerous studies examining its nucleotide sequence variation in a wide range of ethnically diverse populations and determining the role of natural selection in shaping genetic variation at this locus. Several surveys provided clear evidence for a correlation between the prevalence of slow acetylators in human populations and the subsistence strategy adopted by their ancestors in the last 10,000 years, suggesting that a slower rate of acetylation may have provided a selective advantage in populations shifting from foraging to pastoralism/agriculturalism in the Neolithic period [18,20,21,24]. It is thus currently held that the Neolithic transition triggered significant changes in dietary exposure to environmental chemicals that modified the selective regime affecting the NAT2 acetylation pathway. Likewise, and more generally, considering the temporal fluctuations in the kinds and levels of environmentally available xenobiotic compounds over evolutionary time, the metabolic function of NATs is likely to be under adaptive evolution and the *NAT* genes could be broadening or changing substrate specificity in response to changing environmental conditions. Yet, in contrast to the numerous population genetic studies carried out for human polymorphic *NAT* genes, no study to date has investigated the role of positive selection in the expansion and functional diversification of the *NAT* genetic loci at a deeper evolutionary time scale.

The aim of the present work was to thoroughly examine the molecular evolutionary history of the *NAT* gene family during the last ~450 million years. A phylogenetic analysis of 77 *NAT* homologous nucleotide sequences from 38 vertebrate species retrieved from genomic databases was used to determine the likely contribution of gene duplication, gene conversion and positive selection to the evolutionary dynamics of this family. A special emphasis was put on the study of the last ~85 million years of primate *NAT* evolution by determining the *NAT* coding sequences in 13 additional primate species. The results of this work greatly improved our understanding of the evolutionary regime that has shaped the *NAT* protein-coding sequence: it allowed us to clarify the paralogous and orthologous relationships among the *NAT* genes in vertebrate species, date the gene-duplication events giving rise to the human *NAT* gene family, and identify episodes of adaptive evolution at specific sites and domains of the protein.

## Results

### Phylogenetic analysis of vertebrate NAT sequences

To study the molecular evolution of the *NAT* gene family in vertebrates, we retrieved all available *NAT*-homologous sequences from public genomic databases and compiled a dataset of 77 *NAT* coding sequences from 38 species representing all major vertebrate taxa (‘vertebrate dataset’). These 77 sequences included 68 full-length open reading frames (ORFs) with sizes ranging from 864 to 870 bp and 9 partial coding sequences containing 15 to 166 missing nucleotides. In most species, we identified one or two distinct *NAT* sequences but in some others, up to five functional *NAT* genes were found (7, 2 and 1 species harbored 3, 4 and 5 distinct sequences, respectively). A second *NAT* sequence was identified in *Dipodomys ordii* and *Vicugna pacos* but the corresponding ORFs were missing 300 and 500 nucleotides, respectively, so they were not included in the final dataset. No *NAT*-like sequences were found in the current assembly of the dog genome (CanFam2.0), confirming the previous findings of Trepanier and colleagues [25] who were unable to detect *NAT* homologues in any of 25 domestic dogs and 8 wild canids studied. The proportion of sequence identity to human *NAT1* varied from 51.2% (*Danio rerio NAT4*) to 98.9% (*Gorilla gorilla NAT1*) at the nucleotide level. The alignment result of the 77 nucleotide sequences is provided in Additional file 1. Three sequences showed a 3-bp insertion at positions 385–387 resulting in an additional amino acid in the protein (either a phenylalanine in *Ornithorhynchus anatinus NAT1* or a proline in *Gallus gallus NAT2* and *Meleagris gallopavo NAT2*), while eleven sequences from seven species carried a deletion of either 3, 6 or 9 bp, resulting in a loss of either 1, 2 or 3 contiguous amino acids. In all sequences, the three residues composing the ‘Cys-His-Asp’ catalytic triad (Cys68, His107, Asp122), which is essential to the NAT enzymatic activity, were conserved, indicating that the encoded enzymes are likely to be functional.

**Figure 1:**
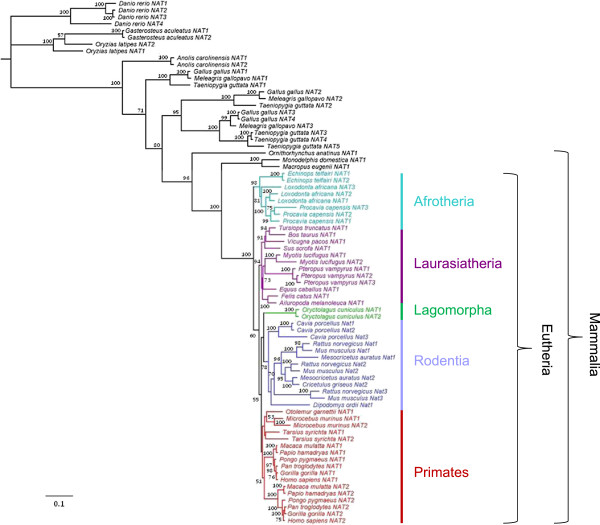
**Phylogenetic tree of the *NAT *gene family based on 77 vertebrate nucleotide sequences.** Shown is the maximum likelihood tree built using PhyML (GTR + I + G) and rooted with the three fish species (*Danio rerio*, *Gasterosteus aculeatus*, *Oryzias latipes*). The bootstrap values of 1,000 replicates are shown as percentages at nodes. Bootstrap values are only shown for nodes with greater than 50% support. The clades of Afrotheria, Laurasiatheria, Lagomorpha, Rodentia and Primates are shown in aqua blue, purple, green, blue and red, respectively.

A maximum-likelihood (ML) phylogeny was constructed with PhyML using the *NAT* coding nucleotide alignment (Figure 1). This phylogeny was largely congruent with the currently accepted phylogeny of vertebrate species at the class (Mammalia, Aves, Reptilia, Actinopterygii) and order (Primates, Rodentia, Lagomorpha, Carnivora, etc.) levels. In most species carrying more than one functional *NAT* gene, the paralogs were more similar to each other than the corresponding orthologs, suggesting multiple species-specific gene duplication events. One exception concerns the sequences of the mouse and rat species which cluster into three paralog groups *Nat1*, *Nat2* and *Nat3*, suggesting that the three genes were present before the divergence of the two rodent species. This may also be the case for the Syrian and Chinese hamsters (*Mesocricetus auratus* and *Cricetulus griseus*, respectively), whose genomes have not been sequenced yet and which may harbor three functional genes as well. Another exception involves the catarrhine species (human, chimpanzee, gorilla, orangutan, macaque and baboon) where a single duplication event probably occurred in the common ancestor of these species giving rise to the human *NAT1* and *NAT2* homologous genes. The tree also suggests that two duplication events prior to the divergence of species also occurred in the Aves clade. Besides these exceptions, our phylogenetic analysis indicates that lineage- and species-specific gene duplications are widespread in the evolutionary history of the *NAT* gene family, so that most *NAT* genes in vertebrates are the products of recent gene duplication events.

However, some aspects of the sequence divergence patterns observed in the tree might also be attributed to concerted evolution *via* interparalog gene conversion which homogenizes the sequences of different members of a gene family within a genome. This would explain why different *NAT* paralogs within a species (e.g., *Cavia porcellus*, *Pteropus vampyrus*, *Myotis lucifugus, Procavia capensis*, *Loxodonta africana*, *Danio rerio*, etc.) cluster together in the tree. To detect and characterize putative recombination events among *NAT* genes, we used six different methods designed to pinpoint recombination break-points and define gene conversion tracts (see Methods). By combining the results of all six methods, a total of eight recombination events were detected affecting seven *NAT* paralog pairs in seven species (Table 1); only four of them were detected by at least two different algorithms. In view of the inferred phylogenetic relationships among *NAT* sequences (Figure 1), the detected conversion events could explain the preferential clustering of *NAT* paralogs in the *Danio rerio*, *Oryzias latipes*, *Echinops telfairi* and *Myotis lucifugus* species. However, when removing all the regions affected by gene conversion from the nucleotide alignment (considering the eight detected conversion events with the largest boundaries), exactly the same topology was obtained in the resulting tree (Additional file 2: Figure S1), with nearly identical bootstrap values (except for an increased statistical support of the clustering of *Oryzias latipes NAT2* with the *NAT1* and *NAT2* sequences from *Gasterosteus aculeatus*; bootstrap value = 909/1000, Additional file 2: Figure S1, instead of 574/1000, Figure 1). This suggests that gene conversion is unlikely to have confounded the analysis and that concerted evolution has only played a minor role (if any) in the patterns of divergence of *NAT* sequences in vertebrates. Overall, our results indicate that the *NAT* gene family has undergone rapid evolution with frequent gene duplications and losses (birth-and-death evolution) throughout vertebrate evolution even among closely related species.

**Table 1:** **Significant recombination events detected among *NAT *genes using six different methods**

**Species**	**Genes**	**5' breakpoint**^**a**^	**3' breakpoint**^**a**^	**Length (bp)**^**a**^	***P*-value range**^**b**^	**Methods**^**b**^
*Rattus norvegicus*	*NAT1* : *NAT2*	382/385/401	609/611	209/225/230	9.06 E-3–1.00 E-6	**GeneConv**, RDP, MaxChi, Chimaera, Bootscan, SiScan
*Oryzias latipes*	*NAT1* : *NAT2*	1	260/262	260/262	8.87 E-3–6.39 E-6	Geneconv, RDP, MaxChi, Chimaera, **Bootscan**
*Echinops telfairi*	*NAT1* : *NAT2*	43	642	600	1.00 E-4	Geneconv
*Myotis lucifugus*	*NAT1* : *NAT2*	600/615	742/749	128/150	6.06 E-4–1.39 E-4	**MaxChi**, Chimaera,
*Mus musculus*	*NAT1* : *NAT2*	382	518	137	2.00 E-4	Geneconv
*Mesocricetus auratus*	*NAT1* : *NAT2*	1	155/163/167	155/163/167	9.32 E-3–2.93 E-4	RDP, Chimaera, **Bootscan**, SiScan
*Myotis lucifugus*	*NAT1* : *NAT2*	1	204	204	3.00 E-4	Geneconv
*Danio rerio*	*NAT2* : *NAT3*	88	507	420	7.90 E-3	Geneconv

^a^ Breakpoint positions refer to the nt positions in the full alignment of the vertebrate *NAT* sequences. They may slightly vary depending on the method used to detect recombinant sequences, leading to different gene conversion tract lengths.

^b^ In cases where multiple methods detected the same or a similar conversion event, we reported the *P*-value range, i.e. the worst and the best *P*-value; the method yielding the best *P*-value is shown in bold. Otherwise, only the *P*-value of the single algorithm detecting a conversion event is presented.

**Figure 2:**
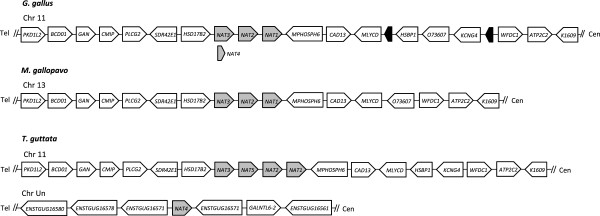
**Gene order and orientation in regions surrounding *NAT *genes on chicken (*Gallus gallus*) chromosome 11, turkey (*Meleagris gallopavo*) chromosome 13 and zebra finch (*Taeniopygia guttata*) chromosomes 11 and Un.** Gene lengths and intergenic distances are not drawn to scale. Grey boxes indicate *NAT*-like sequences. Black boxes represent chicken-specific genes with no homologous sequences in turkey or zebra finch. Double slashes (//) indicate continuing sequence data extending toward the centromeric (cen) and telomeric (tel) parts of the chromosome. In each species, *NAT* genes are arrayed linearly along a single chromosome, indicating an expansion of the *NAT* gene family through tandem repeats, except in zebra finch where a fifth copy is found on an unidentified (Un) chromosome. Chicken *NAT4* (chr11:17,483,758-17,484,630 in galGal3 assembly) represents a short sequence of 873 bp, identical to the *NAT3* protein-coding sequence, which is embedded within the *NAT3* gene (chr11:17,481,972-17,493,917) upstream of the *NAT3* coding sequence.

This scenario is further supported by the fine-scale synteny data retrieved for several vertebrate species in an extended region encompassing the *NAT* genes locus. The dynamic pattern of gene duplication and loss is robustly apparent in comparing the human and mouse genomes, which are especially mature in sequence and annotation (Additional file 3: Figure S2). Although both the human and mouse *NAT* clusters are composed of three loci located in homologous regions of chromosome 8 (two functional genes and one pseudogene in human and three functional genes in mouse), no one-to-one ortholog relationships could be defined because of the presence of a predicted pseudogene not related to *NAT* sequences between the mouse *Nat2* and *Nat3* genes and the total loss of shared synteny on the telomeric side of the clusters. Combined with the phylogenetic relationships inferred in the ML tree (Figure 1), these data rather support a scenario where the human and mouse syntenic gene clusters have originated from a shared ancestral gene or genes, with repeated local gene duplications and losses giving rise to lineage-specific groups of related genes (rodents and primates in the present case). As a further illustration, the genomic sequences of the three bird species studied were similarly compared (Figure 2). An extended region of conserved fine-scale synteny encompasses the chicken (*Gallus gallus*), turkey (*Meleagris gallopavo*) and zebra finch (*Taeniopygia guttata*) *NAT* genes, confirming that the *NAT* paralogs of these species share a common genomic history. Both the chromosomal location and patterns of clustering of avian *NAT* sequences support the existence of three *NAT* genes (corresponding to the *NAT1*, *NAT2* and *NAT3/4/5* clusters of genes in the ML tree) before the split of the three bird species. Thus, the three paralogous *NAT1*, *NAT2* and *NAT3* sequences within each species likely result from the same tandem gene duplication events occurring in the common ancestor to these species. While no further gene duplication event apparently occurred in turkey, additional duplications of ancestral *NAT3* sequences happened independently in the genomes of chicken and zebra finch, giving rise to chicken *NAT4* and zebra finch *NAT4* and *NAT5*, respectively. These data further demonstrate the complex history of duplications of the *NAT* gene family, with many cases of recent gene duplications occurring independently in specific lineages.

### Maximum-likelihood analysis of selective pressures in vertebrate NAT sequences

**Figure 3:**
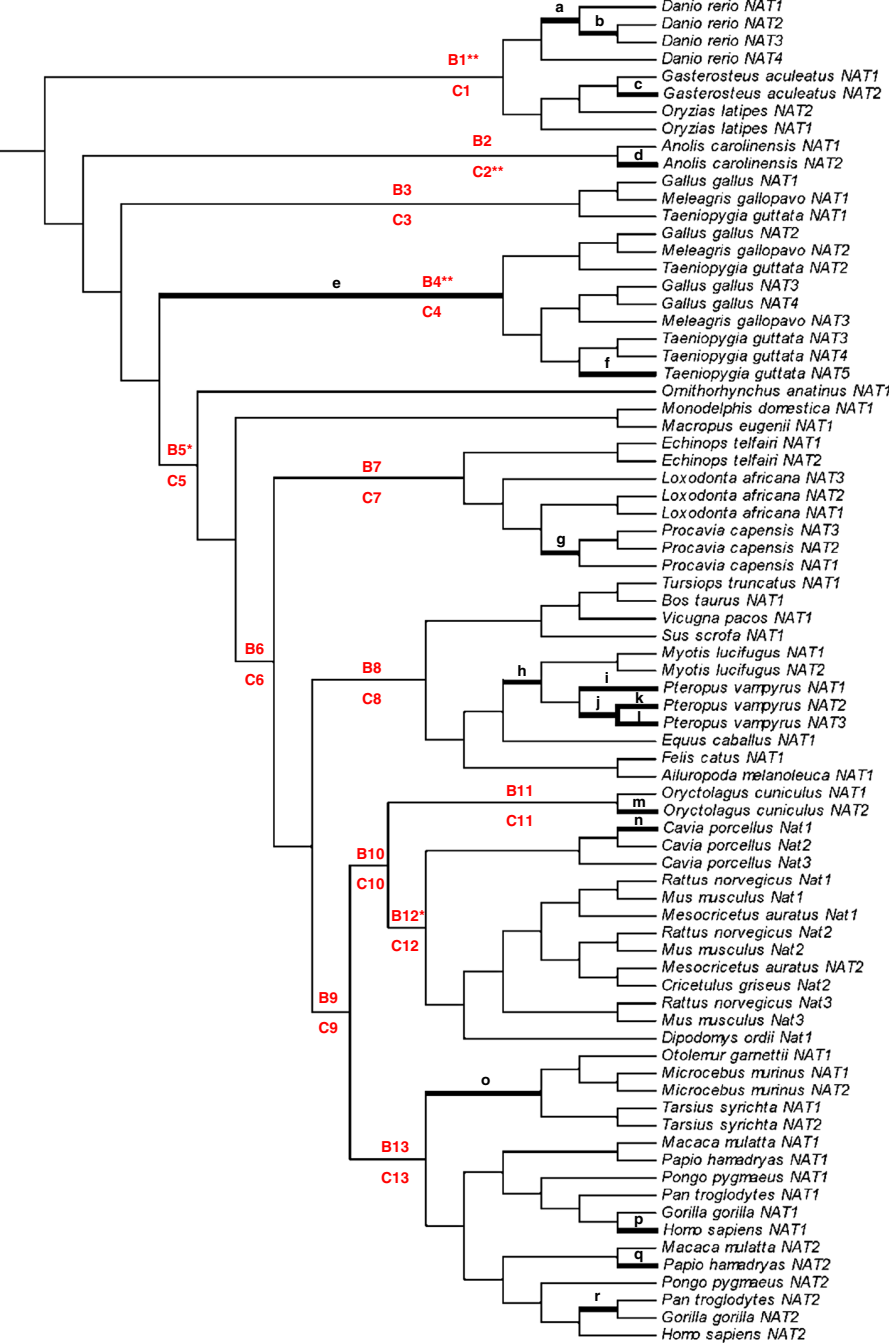
**Evidence for lineage-specific positive selection in the vertebrate *NAT *phylogeny.** The branches with ω ratios > 1, as estimated by the free-ratio model (branch-specific test), are shown with black thick lines and are labeled from a to r. The estimated ω ratio and numbers of nonsynonymous and synonymous changes for each branch are as follows: a (1.26; 56.5/16.3), b (1.76; 22.8/4.7), c (1.07; 6.2/2.1), d (∞; 8.1/0.0), e (231.27; 45.7/0.1), f (1.07; 43.8/15.0), g (1.07; 3.3/1.1), h (1.11; 6.7/2.2), i (2.85; 10.7/1.4), j (2.54; 14.0/2.0), k (∞; 4.9/0.0), l (2.43; 13.4/2.0), m (1.41; 9.2/2.4), n (∞; 8.2/0.0), o (6.31; 12.0/0.7), p (∞; 5.1/0.0), q (1.11; 3.1/1.0), r (1.56; 4.5/1.1). In addition, branch-site tests were carried out to test for positive selection in 13 pre-specified lineages (highlighted in red): the ‘B’ letter refers to the branch-site tests performed at the branch level and the ‘C’ letter refers to those performed at the clade level (multiple branch-site analysis, see Methods). We focused on the major taxonomic groups of the vertebrate phylogeny: Primates (#13), Rodentia (#12), Lagomorpha (#11), Glires (Rodentia + Lagomorpha, #10), Euarchontoglires (Primates + Glires, #9), Laurasiatheria (#8), Afrotheria (#7), Eutheria (#6), Mammalia (#5), Sauropsida (#2), and the fish species (#1). As the group of avian *NAT* sequences was not monophyletic, we separately tested the subgroup of *NAT1* sequences and the subgroup of *NAT2*/*NAT3*/*NAT4*/*NAT5* sequences in birds (#3 and #4, respectively). * Statistically significant branch-site tests at the conventional *P*-value threshold of 0.05 (not corrected for multiple testing). ** Statistically significant branch-site tests at the Bonferroni-corrected threshold of 0.0038 (13 tests).

Since some aspects of *NAT* evolution are likely driven by xenobiotic exposure, we investigated the possible role of natural selection in the evolutionary history of this gene family. To test for evidence of positive selection acting at specific sites of the *NAT* coding region or along particular branches of the phylogenetic tree, a series of nested likelihood ratio tests (LRTs) using different sets of site-specific and branch-specific models were carried out using the codeml program of the PAML package [26] (Table 2). When applied to the DNA codon alignment of the full vertebrate dataset (*n* = 77 sequences), LRTs of site-specific models indicated both varying ω ratio among sites (M0 vs. M3: *P* < 0.0001) and positive selection at a small subset of sites (M2a vs. M1a: *P* = 0.0005; M8 vs. M7: *P* < 0.0001), indicating that the *NAT* coding region has evolved under diversifying selection in vertebrates. Log-likelihood values showed that M8 was the model that best fitted the data. This model showed that 5% of the sites were under positive selection with an average estimated ω ratio of 1.3. The Bayes empirical Bayes (BEB) analysis identified four sites under positive selection with high posterior probabilities (PP) under M8 (PP ≥ 95%): residues 97, 98, 104, and 286, the first three of which are involved in acetyl-CoA (cofactor) binding [27,28]. Likewise, the free-ratio model implemented in PAML, which allows an independent assignment of ω ratios to each evolutionary branch, gave a significantly better fit to the data than the one-ratio model M0, thus suggesting lineage-specificity of ω (*P* < 0.0001; Table 2). Out of the 143 branches of the analyzed phylogeny, 18 branches (i.e. 12.6%) showed evidence of positive selection (ω >1; Figure 3), with the highest ω values for branches d, e, k, n, o, and p (ω ratios ranging from 6.3 to ∞). Interestingly, four branches with evidence of positive selection clustered in the *Pteropus vampyrus NATs* clade (branches i, j, k and l). Detailed analysis of the patterns of nucleotide substitutions along the four branches leading to the three paralogous *NAT* sequences found in this bat species revealed a striking pattern of accelerated amino acid evolution due to positive selection: as many as 39 different nonsynonymous changes affecting the NAT protein-coding sequence occurred within this single clade (Additional file 4: Table S1). It is noteworthy that nearly half (18/39) of these nonsynonymous changes are located either in the interdomain region of the protein, which contains several residues involved in substrate and cofactor binding (codons 204 to 220), or in the C-terminal region, which has a major role in arylamine substrate specificity (codons 267 to 286) [27,29]. Given that positive selection often operates only on a few amino acid sites along particular branches, we employed branch-site models (see Methods) to detect whether some sites along particular branches of the phylogeny are under positive selection. Since the subset of branches to be tested has to be specified *a priori* and independently of the results of the branch-specific test, we decided to focus on those branches defining the major taxonomic groups of the vertebrate phylogeny, numbered from B1 to B13 (Figure 3). Four of these branches, leading to fishes (B1, *P* = 0.0002), avian *NAT2*/*NAT3*/*NAT4*/*NAT5* sequences (B4, *P* = 0.0015), mammals (B5, *P* = 0.049) and rodents (B12, *P* = 0.048), showed evidence of positive selection at the non-corrected threshold of 0.05. Only the two formers remained significant after correction for multiple testing. Two sites were identified by the BEB analysis as positively selected (PP ≥ 95%): codon 11 (PP = 0.958) along the lineage leading to avian *NAT2*/*NAT3*/*NAT4*/*NAT5* sequences and codon 272 (PP = 0.957) along the lineage leading to rodents. When branch-site tests were performed at the clade level (C1 to C13), only lizard sequences provided significant results (C2, *P* = 0.0004), which persisted after correction for multiple testing. In this clade, two codons were identified as positively selected: 100 (PP = 0.960) and 102 (PP = 0.964). Note that the four sites identified by the branch-site tests (11, 272, 100 and 102) are in addition to those identified with site-specific models.

**Table 2:** Results of PAML analyses for the vertebrate dataset

**Data set**	**2Δln*L***	**df**	***P-*value**	**Proportion of sites with ω > 1 (average ω for these sites)**	**Positively selected codons**^**a**^
**All vertebrates (*n*** **= 77)**					
M1a vs. M2a	15.22	2	***P*** **= 0.0005**	0.017 (ω = 2.0)	
M7 vs. M8	34.89	2	***P*** **< 0.0001**	0.050 (ω = 1.3)	97**, 98*, 104*, 286*
M0 vs. free ratios	510.67	142	***P*** **< 0.0001**		
**Mammals (*n*** **= 55)**					
M1a vs. M2a	31.27	2	***P*** **< 0.0001**	0.027 (ω = 2.3)	
M7 vs. M8	47.39	2	***P*** **< 0.0001**	0.066 (ω = 1.6)	97**, 98*, 104*, 214*, 286**
M0 vs. free ratios	265.71	100	***P*** **< 0.0001**		
**Primates (*n*** **= 17)**					
M1a vs. M2a	0	2	*P* = 1.0	none	
M7 vs. M8	0	2	*P* = 1.0	none	
M0 vs. free ratios	36.45	28	*P* = 0.13		

*n*, number of sequences; 2Δln*L*, twice the log-likelihood difference of the models compared; df, degrees of freedom; *P*-value, level of significance (*P*-values < 0.05 are shown in bold).

^a^ Sites pinpointed to be under positive selection under the selection model with the highest likelihood (M8 for the vertebrate and mammalian datasets) by Bayes Empirical Bayes (BEB) analysis. Only sites with posterior probability greater than 0.95 (*) or 0.99 (**) are shown. Sites are numbered according to the full human coding sequence.

When restricting the analysis to the 55 mammalian sequences of the vertebrate data set, similar results were obtained. Model M8 provided the best fit to the data with 6.6% of sites having a positive ω of 1.6 (Table 2). The same codons were pinpointed to be under positive selection with PP ≥ 95%, plus an additional site located in the acetyl-CoA binding pocket (codon 214). However, when the analysis was restricted to the 17 primate sequences, no significant evidence of positive selection was detected (*P* > 0.05), neither at the site nor at the branch level. This suggests either that the selective pressure that recurrently drove positive selection of vertebrate *NAT*s became relaxed in the primate lineage, or that the statistical power provided by the small number of sequences available for analysis may be insufficient to get significant results when performing the LRT tests implemented in PAML. These tests have indeed been shown to lack power when data contain only few numbers of slightly divergent sequences [30]. In such a case, sampling a larger number of lineages is considered as the best way to improve the accuracy and power of LRTs and has been shown to cause a spectacular rise in power, even when sequence divergence is low [30,31]. Therefore, we sought to gain power by determining the *NAT* coding sequences in 13 additional primate species, bringing up to 22 the number of species available for analysis in the primate clade.

### NAT sequence evolution and analysis of selective pressures in the primate lineage

A more thorough investigation of the evolutionary history of the *NAT* gene family was conducted in primates by extending the analysis to 13 additional primate species (including 3 hominoids, 8 Old World and 2 New World monkeys) in which *NAT* nucleotide sequences were determined for the first time. Two functional *NAT* genes were found in all 13 species, whereas a third *NAT* sequence, abolished by frameshift mutations and highly similar to the human pseudogene *NATP*, was found in ten of them. In all, the full primate data set comprised 59 nucleotide sequences from 22 primate species, including 17 database sequences and 42 newly generated ones. Whereas functional sequences were all of the same length (a full *NAT* ORF of 870 bp), pseudogene sequences displayed considerable length variation due to the significant number of indels disrupting *NAT* ORF (ranging from 865 bp for *Colobus guereza NATP* to 881 bp for *Gorilla gorilla NATP*), resulting in a multiple alignment of 890 sites (Additional file 5). These indels involve both single and multiple nucleotides. All *NATP* sequences have in common a single 1-bp deletion in position 77, which is likely to be the first frameshift mutation that occurred in the ancestral *NATP* coding sequence.

**Figure 4:**
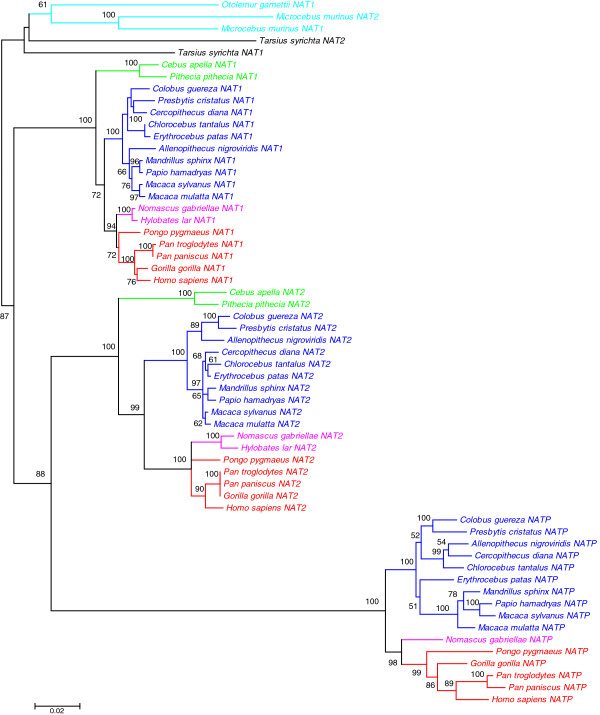
**Phylogenetic tree of the *NAT *gene family in primates.** Shown is the maximum likelihood tree built using PhyML (GTR + I + G) with the multiple alignment of 59 *NAT* nucleotide sequences from 19 distinct primate species. The tree was rooted with the non simian species (*Otolemur garnetti*, *Microcebus murinus*, *Tarsius syrichta*). The bootstrap values of 1,000 replicates are shown as percentages at nodes. Bootstrap values are only shown for nodes with greater than 50% support. The clades of Strepsirrhini, Platyrrhini, Cercopithecidae, Hylobatidae and Hominidae are shown in turquoise, green, blue, pink and red, respectively.

The ML tree built using PhyML support monophyletic Strepsirrhini, Simiiformes (Anthropoidea), Platyrrhini, Catarrhini, Cercopithecoidea and Hominidea clades (Figure 4). In Simiiformes, *NAT* genes clearly cluster into three paralog groups, all strongly supported (bootstrap values = 1000/1000) and regrouping sequences orthologous to human *NAT1*, *NAT2* and *NATP*. The paralog group of *NAT1* sequences displays the highest level of nucleotide sequence identity (mean pairwise value 96.7%, range 93.2%–99.8%), followed by *NAT2* (mean 95.1%, range 91.2%–100%) and *NATP* (mean 93.3%, range 89.7%–98.9%). The accelerated evolution of the *NATP* pseudogene is clearly apparent from the long branch leading to this clade, probably reflecting relaxed purifying selection subsequent to the loss of protein functionality. Contrary to the two functional genes, *NATP* sequences were obtained only in catarrhine species. Hence, our phylogenetic analysis suggests that the three human *NAT* genes emerged from a first duplication event in the common ancestor of Simiiformes, yielding *NAT1* and an ancestral *NAT* gene which in turn, duplicated in the common ancestor of Catarrhini, eventually giving rise to *NAT2* and *NATP*. The absence of *NATP* in one of the Hylobatidae species (*Hylobates lar*) can be explained either by the loss of this gene (or of the primer target regions) in this species or by a substantial divergence of this sequence from its related orthologs, hampering its effective amplification. Obviously, we cannot exclude the possibility that similar reasons may explain the absence of *NATP* in platyrrhine species. However, the high number of trials performed (11 different primer pairs tested, arranged on different parts of the gene including the 5’ and 3’ regions, and varying PCR conditions) make this possibility unlikely and rather support a true absence of the pseudogene in Platyrrhini and *Hylobates lar*.

The phylogenetic analysis also demonstrates that *NAT1* and *NAT2* paralogs found in *Microcebus murinus* and *Tarsius syrichta* are the result of independent gene duplication events that occurred after the divergence of these species from the simian lineage and after the divergence of Strepsirrhini and Tarsiiformes. Because no gene conversion was detected with any of the six recombination detection methods for any pair of sequences in the primate dataset, we can exclude concerted evolution as a possible explanation for the greater similarity of *NAT* paralogs within *M. murinus* and *T. syrichta* species. *Otolemur garnetti* is the only primate species with a single *NAT* gene identified. However, genomic coverage is still limited (2-fold) for this species and we cannot exclude that additional *NAT* copies may exist in its genome.

**Figure 5:**
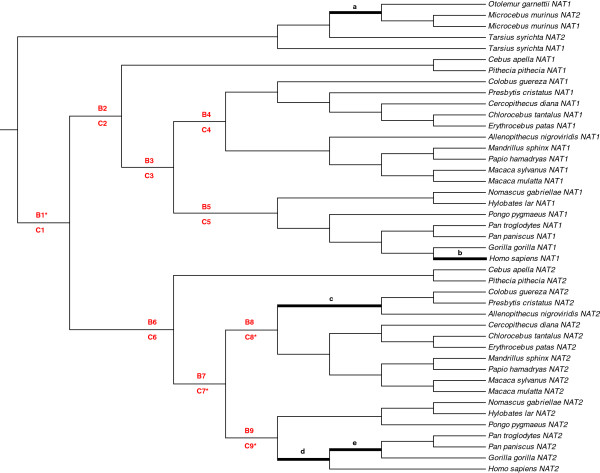
**Evidence for lineage-specific positive selection in the primate *NAT *phylogeny.** Branches with evidence of positive selection (ω > 1), as estimated by the free-ratio model (branch-specific test), are shown with black thick lines and are labeled from a to e. The estimated ω ratio and numbers of nonsynonymous and synonymous changes for each branch are as follows: a (∞; 8.1/0.0), b (∞; 5.1/0.0), c (∞; 7.0/0.0), d (1.07; 3.9/1.3), e (1.63; 4.5/1.0). In addition, branch-site tests were carried out to test for positive selection in 9 pre-specified lineages (highlighted in red): the ‘B’ letter refers to the branch-site tests performed at the branch level and the ‘C’ letter refers to those performed at the clade level (multiple branch-site analysis, see Methods). *Statistically significant branch-site tests at the conventional *P*-value threshold of 0.05 (not corrected for multiple testing). **Statistically significant branch-site tests at the Bonferroni-corrected threshold of 0.0056 (9 tests).

Next, we investigated the role of positive selection in the evolution and diversification of the *NAT* gene family in primates by applying PAML codon-based models to the 43 coding sequences of the primate data set (16 pseudogene sequences excluded) (Table 3). Despite the increased power afforded by the larger number of sequences included in the analysis (43 instead of 17 in the initial analysis), we did not find any evidence of adaptive selection during primate evolution at any *NAT* coding site. Models M1a and M2a showed no difference (2Δln*L* = 0), and model M8 was not significantly more likely than model M7 (2Δln*L* = 3.49, *P* > 0.05). Detailed analysis of the tree using the free-ratio model of codeml did, however, identify a number of branches with ω values higher than 1.0, the strongest evidence being found in the lineage leading to Strepsirrhini (branch a, Figure 5), consistent with the full vertebrate dataset analysis results. The branch-site tests, performed at both the branch and clade levels (Figure 5), identified one branch evolving at an elevated rate compared to other branches of the phylogeny (B1, *P* = 0.011) as well as three clades within the set of orthologous *NAT2* sequences with evidence of positive selection (C7, *P* = 0.019; C8, *P* = 0.040; C9, *P* = 0.017, C8 and C9 being component clades of C7). These results were, however, no longer significant after applying the Bonferroni correction for multiple comparisons. No positively selected sites were identified with significance (PP ≥ 95%) with these branch-site tests.

**Table 3:** Results of PAML analyses for the primate dataset

**Data set**	**2Δln*L***	**df**	***P-*value**	**Proportion of sites with ω > 1 (average ω for these sites)**	**Positively selected codons**^**a**^
**All *NAT *coding sequences (*n*** **= 43)**					
M1a vs. M2a	0	2	*P* = 1.0	none	
M7 vs. M8	3.49	2	*P* = 0.17	none	
M0 vs. free ratios	114.89	80	***P*** **= 0.006**		
**Orthologous sequences to human *NAT1 *(*n*** **= 19)**					
M1a vs. M2a	0	2	*P* = 1.0	none	
M7 vs. M8	0	2	*P* = 1.0	none	
M0 vs. free ratios	49.09	34	***P*** **= 0.04**		
**Orthologous sequences to human *NAT2 *(*n*** **= 19)**					
M1a vs. M2a	8.48	2	***P*** **= 0.01**	0.038 (ω = 4.8)	
M7 vs. M8	11.24	2	***P*** **= 0.004**	0.041 (ω = 4.6)	191*, 173*
M0 vs. free ratios	27.52	34	*P* = 0.77		

*n*, number of sequences; 2Δln*L*, twice the log-likelihood difference of the models compared; df, degrees of freedom; *P*-value, level of significance (*P*-values < 0.05 are shown in bold).

^a^ Sites pinpointed to be under positive selection under the selection model with the highest likelihood (M8 for the set of *NAT2* orthologous sequences) by Bayes Empirical Bayes (BEB) analysis. Only sites with posterior probability greater than 0.95 (*) or 0.99 (**) are shown. Sites are numbered according to the full human coding sequence.

A possible limitation of our analysis is that it relies on the assumption that the different orthologous and paralogous sequences compared have equivalent functions. The inclusion in the alignment of homologous proteins with different biological functions which may have experienced different selective regimes can indeed give rise to incorrect estimations of ω. This could be the case if, as observed for the human *NAT1* and *NAT2* paralogs, the two ancestral *NAT1* and *NAT2* paralogs diverged in function soon after the duplication event, before the first speciation leading to Catarrhini and Platyrrhini. Thus, in order to maintain a correct comparative framework and to avoid the inclusion of paralogous sequences where changes in protein function occurred, we conducted separate analyses of the sets of orthologous *NAT1* sequences and orthologous *NAT2* sequences found in Simiiformes.

For the data set including the 19 sequences orthologous to human *NAT1*, models incorporating positive selection (M2a and M8) provided no better fit to the data than models including only purifying selection and neutrally evolving sites (M1a, M7) (Table 3). The null model M0, which assumes only one ω for all amino acid sites, could not be rejected (*P* > 0.05). Under this model, the estimate of ω was 0.19, indicating that *NAT1* sequences have been subject to strong purifying selection to maintain protein function over time. The selective regime has been variable among species since different ω values along different branches were significantly different from each other (*P* = 0.04). Interestingly, despite the dominant role of purifying selection in NAT1 protein evolution, a recent episode of positive selection was detected specifically and exclusively in the human lineage (branch b with ω = ∞; Figure 5). Five codon sites were found to show nonsynonymous substitutions along this branch (E46D, I149V, E195K, A214S, E276Q), whereas no synonymous codon changes occurred.

By contrast, site-specific codeml analysis performed on the set of sequences orthologous to human *NAT2* yielded very different results with a rather strong evidence of positive selection (M2a vs. M1a: *P* = 0.01; M8 vs. M7: *P* < 0.004, Table 3), indicating that NAT2 has been subject to diversifying selection to change its amino acid sequence over time (Table 3). Parameter estimates under model M8, which provided the best fit to the data, suggested 4.1% of sites to be under positive selection with ω = 4.6. Estimates from posterior probability provided significant support of positive selection for codons 173 and 191 (PP ≥ 95%) under M8. It is noteworthy that these two codons were also identified as positively selected with PP ranging from 77% to 87% in the three *NAT2* clades pointed out by multiple branch-site analysis (C7, C8 and C9, Figure 5). Codon site 129, which plays a key role in determining NAT2 substrate selectivity, was identified with lower confidence (PP ≥ 90%). Although the free-ratio model was not a better fit to the data than a single ω ratio for the entire *NAT2* phylogeny (2Δln*L* = 27.52, *P* > 0.05), some branches did exhibit ω > 1 (branches c, d and e; Figure 5).

### Variation of selective pressures along the NAT protein sequence

**Figure 6:**
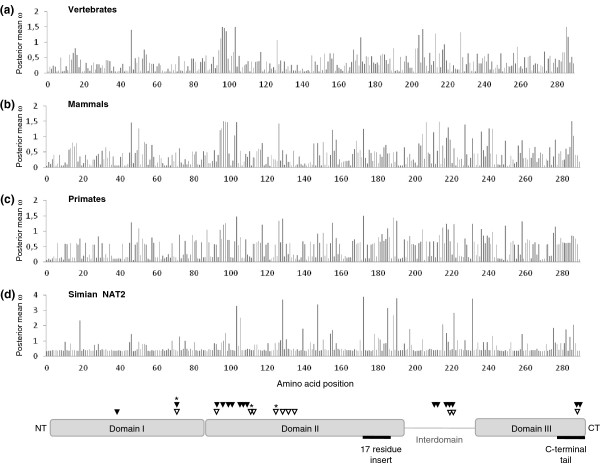
**Variable selective pressures (ω) along NAT protein sequence.** The posterior mean of ω was estimated for each of the 290 amino acid residues by the codeml program (**a**) in the vertebrate dataset (*n* = 77 coding sequences), (**b**) in the mammalian dataset (*n* = 55 coding sequences), (**c**) in the primate dataset (*n* = 43 coding sequences), (**d**) in the set of 19 simian NAT2 coding sequences. A graphical representation of the three-domain structure of human NAT1 and NAT2 proteins is shown at the bottom. Residues 1–83 in the N-terminus form domain I, mainly consisting of α-helices; residues 84–192 form domain II, mainly consisting of β-strands, followed by inter-domain comprising residues 193–229, and residues 230–290 consisting of both α-helix and β-strands form domain III. The amino and carboxyl termini are labeled as NT and CT, respectively. The 17-residue insertion and carboxyl-terminal tail of human NATs, which are lacking in the structures of prokaryotic NATs, are highlighted with thick lines. Residues of the catalytic triad are highlighted with stars, residues interacting with CoA are highlighted with black triangles, and residues involved in substrate binding are highlighted with white triangles.

The three-dimensional crystal structure of NAT isoenzymes has been resolved for several organisms, enabling a thorough evaluation of structure-function relationships without the limitations inherent to molecular homology modeling [5]. The fold of human NAT1 and NAT2 proteins largely resembles the overall structure of their prokaryotic orthologs, traditionally described as composed of three domains, each of which being approximately equal in length [27] (Figure 6). The first domain is predominantly α-helical, the second domain mainly consists of β-strands and the third domain is a α/β lid. The first two domains, which have been implicated in active-site pocket formation and acetyl-CoA binding, are connected to the third domain through the α-helical interdomain. The third domain has been associated with acetyl-acceptor specificity [27,32]. In human NATs, residues 167 and 183 bracket a 17-residue insertion, which is absent in the structures of prokaryotic NATs. This loop located in domain II is associated with the domain III β-sheet, apparently providing structural stability and limiting active site access [33]. Also, the C-terminal tail is not α-helical, as is the case for the bacterial NATs, but is instead a coil that reaches around and associates with the active site cleft, thereby playing a key role in defining the size and shape of the active site pocket.

The well-known relationship between the primary sequence of eukaryotic NATs and the structural and functional features of these enzymes prompted us to examine the variation of ω along the NAT protein sequence in the different datasets investigated: (i) the vertebrate dataset, including the full set of 77 vertebrate sequences as well as a subset of 55 mammalian sequences, and (ii) the primate dataset, including the full set of 43 primate sequences as well as the subset of 19 simian NAT2 orthologous sequences. We looked at the variation of the posterior mean of ω as estimated by the codeml program under the site-specific model that best fitted the data (Figure 6). Moreover, we tested for significant differences in the mean ω value between different parts of the protein (domain I, domain II, interdomain, domain III, 17-residue insert, C-terminal tail) and different sets of sites (residues involved either in CoA binding or in substrate binding, Additional file 6: Table S2 and Additional file 7: Table S3). Our results revealed a significant heterogeneity of ω between the four protein domains (domain I, domain II, interdomain, and domain III) in the four datasets analysed (Kruskal-Wallis *P* values ≤ 0.03). More specifically, the first domain was found to evolve under more selective constraints, with a mean ω value being significantly lower than those observed for domain II, domain III or the rest of the sequence (Mann-Whitney *P* < 0.05, Additional file 6: Table S2 and Additional file 7: Table S3). By contrast, domains II and III did not show any differences in their mean ω value when compared to the rest of the sequence (Mann-Whitney *P* > 0.05). These results were consistently observed in the four datasets examined. Besides, the interdomain region, 17-residue insert and C-terminal tail were all found to evolve in vertebrates under more relaxed selective constraints than the rest of the sequence, as reflected by significantly higher ω values (Mann-Whitney *P* = 0.03, *P* = 0.02, *P* = 0.007, respectively). A similar finding was observed for the C-terminal tail in the mammalian (*P* = 0.06), primate (*P* = 0.01) and simian NAT2 (*P* = 0.03) datasets, consistent with the major role of this region in determining arylamine substrate specificity. Interestingly, the mean ω value of the set of sites involved in CoA binding was strikingly high when compared to the mean ω value at the global protein level in vertebrates (0.70 vs. 0.34, Mann-Whitney *P* = 0.002), mammals (0.77 vs. 0.40, *P* = 0.003) and primates (0.73 vs. 0.47, *P* = 0.011). Conversely, adaptive molecular evolution was evidenced in the simian NAT2 dataset for those sites involved in substrate binding compared to the full set of sites in the protein (1.31 vs. 0.66, *P* = 0.044).

## Discussion

The general role of NATs in the detoxification and metabolic activation of aromatic amine xenobiotics, ranging from dietary components to common environmental toxins and pharmaceuticals, has been well documented (reviewed in [34-36]). The possibility that the NAT enzymes could be broadening or changing their substrate specificity in accordance to the high diversity of xenobiotics compounds environmentally available suggests that their metabolic function could be under adaptive evolution and makes them a promising subject for evolutionary analyses. Numerous studies have indeed identified the signature of different selective pressures in genes involved in the metabolism of exogenous substances [37-44]. However, in contrast to the evolutionary processes affecting the *NAT2* gene in humans, which have been the subject of extensive research, little is known about the role of molecular adaptation in the evolution and diversification of the *NAT* gene family as a whole on a longer evolutionary time scale.

The ongoing sequencing of entire genomes from various organisms is providing unparalleled opportunity to trace the evolutionary history of the *NAT* gene family by enabling the inference of the phylogenetic relationships among *NAT* sequences over a wide range of taxa. Several previous surveys of public genomic databases have retrieved *NAT*-like sequences and documented the distribution of *NAT* genes across all major clades of life [1,2,45]. The last survey by Glenn et al. [2] provided an exhaustive annotation of *NAT*-homologous sequences recovered through inspection of 2445 genomes encompassing all major taxa from bacteria and archaea to protists and fungi, to animals. The same investigators also performed phylogenetic analyses of the retrieved NAT-homologous protein sequences, providing a broad perspective of NAT evolution. Another study, focusing specifically on the phylogeny of *NAT*s in fungi, has also been published since then [3]. In the present study, we expanded the previous survey by Glenn et al. [2] by performing a comprehensive search of *NAT*-like sequences in the genomes of vertebrate species and compiled an up-to-date dataset of 77 vertebrate *NAT* sequences from 38 distinct species, among which 26 were identified for the first time (Figure 1). In contrast to the previous dataset of 55 vertebrate sequences in Glenn et al. [2], we decided to exclude one lizard and three fish sequences from analysis (*Anolis carolinensis NAT3*, *Oryzias latipes NAT3*, *Fugu rubripes NAT1*, *Tetraodon nigroviridis NAT1*) because they aligned poorly with other vertebrate *NAT*s and introduced too many gaps and alignment ambiguities. The deduced protein sequence of these four genes clustered together in a monophyletic clade with three invertebrate NAT protein sequences at the basal position of the vertebrate NAT phylogeny reconstructed by Glenn et al. [2]. The authors suspected the possibility that this clustering might be an artifact stemming from long branch attraction, supporting our decision to exclude these particular sequences from our phylogenetic analysis of vertebrate *NAT* genes. It is also important to note that, because the vertebrate genomes we considered are in various stages of draft sequencing, assembly, and annotation, we cannot exclude that the present survey missed additional extant *NAT* sequences. This warrants some caution in the conclusions that may be drawn from the present phylogenetic analysis. The increased availability of genome sequence data from diversified taxa is likely to continue to improve our understanding of the evolutionary history of the *NAT* gene family in vertebrates.

Our phylogenetic analysis of vertebrate *NAT* sequences nevertheless demonstrated that the *NAT* gene family has evolved under a dynamic process called birth-and-death evolution. This process, which operates through three major mechanisms - neofunctionalization, subfunctionalization, and pseudogenization, is thought to be an important source of genetic diversity and evolutionary change that affords functional diversification over short timescales [46]. Our results are in accordance with the previous observations by Thomas [41] that genes encoding enzymes that function as xenobiotic detoxifiers are often phylogenetically unstable genes that undergo rapid birth-and-death evolution, possibly in response to changing environmental conditions. It is noteworthy that Martins et al. [3] found similar patterns of gain and loss in fungi, arguing for a complex dynamics of these genes in a broader range of organisms.

Concerted evolution via interlocus gene conversion is increasingly recognized as a major feature of evolution in small multigene families (e.g., [37,47-49]). By homogeneizing the sequence of paralogous gene copies, the converted paralogs may come to resemble one another more than they do to orthologous sequences in other species. Therefore, gene conversion may potentially alter the relationships among paralogs and lead to the conclusion of independent duplications instead of multiple gene conversions in multiple species. The work carried on the CYP1A1 subfamily [37] provides a good example of how gene conversion can obfuscate gene orthology relationships and lead to incorrect conclusions when based solely on the results of traditional phylogenetic analyses. However, identification of gene conversion events remains a challenging task as the methods commonly employed for detecting such events can have a high false-negative rate, particularly when gene conversion is frequent and covers a large portion of the duplicates [50]. It is thus advocated to consider the occurrence of gene conversion using various recombination detection algorithms and to combine information coming from both phylogenetic analysis and fine-scale synteny maps. We have used six different methods to evidence possible recombination events between paralogous pairs. Only four events, each involving a *NAT1*-*NAT2* pair, were detected by at least two methods (one each in the fish *Oryzias latipes* and rat *Rattus norvegicus*, and two in the bat *Myotis lucifugus*, Table 1), thus suggesting that concerted evolution has played only a minor role in the diversification of the vertebrate *NAT* gene family. This conclusion is further supported by: (i) the identical phylogenetic relationships inferred from *NAT* sequences of which the regions putatively involved in gene conversion events were truncated (Additional file 2: Figure S1), and (ii) the fine-scale synteny comparisons between the three avian species analyzed (Figure 2), as well as between human and mouse (Additional file 3: Figure S2), which evidenced multiple gene duplications events occurring independently in specific lineages. Thus, our analysis of vertebrate *NAT* sequence data suggests that gene conversion is unlikely to have played a major role in the patterns of divergence of *NAT* sequences and rather supports a scenario of multiple independent gene duplications.

In humans, there are two NAT isoenzymes encoded at two polymorphic loci. *NAT2* polymorphisms modify individual cancer risk and drug response, or susceptibility to adverse drug reactions [15,16,51]. Although less well-established, human *NAT1* also exhibits genetic polymorphism and several-albeit as yet inconclusive-studies have suggested that variant *NAT1* genotypes are associated with susceptibility to a number of diseases including various cancers [15] and birth defects [52-56]. Given their high level of polymorphism and their association with variable responses to environmental toxins and drugs, these genes are good candidates to test for positive selection and several studies have investigated the possible role of natural selection in shaping genetic variation at these loci (e.g., [17,23] etc.). Besides their role in phase II metabolism of xenobiotics, several studies have explored the possible endogenous roles of these enzymes. While no endogenous substrate has been identified to date for NAT2, several lines of evidence support the role of human NAT1 in folate catabolism, thereby providing an explanation for its postulated association with congenital defects linked to a disruption in folate metabolism [6]. The widespread tissue distribution of NAT1 and its early expression in development further support a physiological role of this enzyme which might be essential to normal embryonic development (see [10] for review). Therefore, since *NAT1* and *NAT2* appear to have distinct functional roles in humans, it is worthwhile to investigate whether these genes have experienced different selective regimes throughout evolution. The identification of the true orthologous sequences to human *NAT1* and human *NAT2* in Simiiformes and their determination in 13 additional simian species (Figure 4) provided us the necessary power to enable a separate analysis of the two sets of sequences and evaluate the changes in selective pressures experienced by the two gene copies after their functional divergence. Interestingly, two distinct evolutionary patterns emerged for the two paralogs (Table 3). Our analysis suggested a dominant role of purifying selection in NAT1 protein evolution, acting for a conservation of biochemical functions which is in agreement with the role of NAT1 in endogenous metabolism and homeostasis. Note, however, that a signal of positive selection was detected in the human lineage for this gene and that the pattern of evolution of *NAT1* is likely to be mosaic with some evidence of positive selection in certain lineages (in humans but maybe also in several other species not included in the present analysis). By contrast, in most of the species investigated, NAT2 was predicted to evolve under diversifying selection to change its amino acid sequence over time, probably in response to changes in xenobiotic exposure. This finding is consistent with the observations made at a population level within the human species by previously published studies which supported an adaptive evolution of the *NAT2* gene through either balancing or directional selection [17,18,20,21,23,24]. Similarly, the low level of polymorphism reported at *NAT1* within the human species [17,23] is consistent with the action of purifying selection evidenced at the interspecies level in the present study. Evolutionary analyses thus strongly support a differential role of the two isoenzymes and the involvement of NAT1 in endogenous metabolic pathways. Although the folate catabolite pABG is the only endogenous substrate identified to date, one cannot dismiss the possibility that other as yet unknown endogenous substrates and physiological roles may exist for NAT1.

Besides an evaluation of the selective forces acting on members of the *NAT* gene family, our ML-based phylogenetic analysis allowed us to estimate the strength of natural selection acting at a codon level and to shed light on episodes of adaptive evolution at specific sites and domains of the protein. Several amino acid sites were predicted to be under positive selection with high PP (PP ≥ 95%) throughout vertebrate evolution: codons 97, 98, 104, 214, 286. Interestingly, four of these codon positions were shown to be involved in CoA binding according to the recent structural studies performed on human NAT1 and NAT2 isoenzymes [27]. The residues 97P and 98 V are involved in hydrophobic interactions with the adenine ring of CoA, whereas the amide nitrogen of residue 104 G and the hydroxyl group of 214 T form hydrogen bonds with the pyrophosphate group of CoA. It is noteworthy that the fifth codon position predicted to be under adaptive evolution is the site of a well-known polymorphism in the human NAT2 protein: G286E (c.857 G > A), which defines the NAT2*7 slow haplotype series, is one of the four major nonsynonymous substitutions encountered in human populations and is particularly common in Asia [24]. Functional studies of the G286E variant in mammalian cells demonstrated reduced affinity to both substrate and cofactor acetyl-CoA, resulting in reduced catalytic activity towards some substrates (such as sulfamethazine and dapsone) but not others (such as 2-aminofluorene and isoniazid) [57]. Codon 286 is located on the C-terminal tail in the third domain directly adjacent to the active site. Because the C-terminal tail has an important role in defining the size and shape of the active site cavity, a significant amino acid change at this position is likely to alter active site access and substrate selectivity, which is consistent with the substrate-dependent activity changes observed experimentally for the G286 variant. Such a significant change to a C-terminal residue adjacent to the active site is also likely to affect acetyl-CoA binding and 68C acetylation [58]. Note that the branch-site tests identified four additional sites (11, 100, 102 and 272) as having evolved under positive selection along particular lineages of the vertebrate phylogeny or in a specific clade (B4, B12 and C2, Figure 3). Interestingly, codon 102 is also known to be involved in CoA binding and codon 272 is located in the C-terminus region of the protein. Moreover, two codon sites (173 and 191) were pinpointed to be under diversifying selection throughout simian NAT2 evolution. While the functional and/or structural significance of these two codons have not yet been explored, they are likely to be important in the function of the NAT2 protein and further investigations are warranted to define their potential relevance. In contrast to these positively selected codons, very low values of ω, suggesting rather strong functional constraints, were observed for the three sites of the catalytic triad: 0.054, 0.051 and 0.050 for Cys68, His107, Asp122, respectively, in the vertebrate dataset. These three sites were all conserved in the entire set of NAT coding sequences considered, thereby confirming the importance of these three residues in the activation of the active site cysteine residue. Interestingly, domain I was found to be more constrained than the other domains of the protein, consistent with the lower variability displayed by this region (mean Shannon entropy (*H*) = 0.70) as compared to domain II (*H* = 0.84) and domain III (*H* = 0.93). Conversely, higher ω values were observed for the interdomain, 17-residue insert and C-terminus regions of the protein. In particular, the C-terminal tail was shown to evolve under more relaxed selective constraints in the four datasets examined and many sites pinpointed to be under adaptive evolution were located in this region. This is consistent with the association of this region with different acetyl-CoA binding properties and substrate specificities.

## Conclusions

In this study, we provided unequivocal evidence that the *NAT* gene family has undergone a complex history of duplications and possibly gene losses, as observed for many multigene families encoding xenobiotic-metabolizing enzymes (e.g., [39-41,43]). We also found evidence of positive selection for amino acid change in the NAT protein sequence in many lineages throughout vertebrate evolution, suggesting that diversification of the *NAT* gene family is achieved by a combination of gene duplication and selection-driven divergence in sequence. By then focusing on the evolution of this family in primates, we revealed different selective regimes for the two primate paralogs *NAT1* and *NAT2*, consistent with a differential role of the two isoenzymes. Our evolutionary analyses strongly support the key role of the C-terminal region which should be a primary focus of future functional studies.

## Methods

### Collection of vertebrate NAT sequences from genomic databases

All available vertebrate genomes in various stages of draft sequencing, assembly, and annotation were searched for the presence of putative *NAT* genes by TBLASTN searches in the National Center for Biotechnology Information (http://www.ncbi.nlm.nih.gov) and Ensembl (http://www.ensembl.org) databases, using the protein sequence of human NAT1 as the query and an *E*-value cut-off of 10^-5^. Once we identified the first set of vertebrate sequences using the human probe, subsequent searches were conducted by using the sequences of representative species within a given taxonomic class or order to identify additional sequences in the same group of taxa. More specific searches were also performed using the *NAT* protein-coding sequence identified in one organism to look for additional *NAT* paralogs in its genome. We discarded sequences with more than 200 missing nucleotides and only sequences with an identified ORF were kept in the final data set. Sequences from the Syrian hamster (*Mesocricetus auratus*) and the Chinese hamster (*Cricetulus griseus*) were obtained from GenBank (accession numbers: U05271 *Mesocricetus auratus Nat1*, L24912 *Mesocricetus auratus Nat2*, NM_001244413 *Cricetulus griseus Nat2*). Fine-scale synteny data around the *NAT* genes locus were retrieved from Genomicus v63.01 [59] and the UCSC genome browser [60].

To name the *NAT* sequences identified in vertebrate species, we followed the official gene nomenclature for non-human *NAT* genes in eukaryotes and prokaryotes (http://mbg.duth.gr/non-humannatnomenclature/): names were given according to the descending identity between each reported *NAT* sequence and human *NAT1*. The symbol *NAT1* was thus assigned either to the *NAT* sequence demonstrating the highest identity to human *NAT1* or to the only *NAT* gene found in a specific genome. In rodents, the nomenclature followed the consensus for mouse *Nat* genes, with rodent *Nat2* representing the sequence bearing higher identity to human *NAT1* and rodent *Nat1* being more similar to human *NAT2*.

### Amplification, cloning and sequencing of NATs in primates

Based on available DNAs, we selected a representative number of primate specimens belonging to 13 distinct genera, including 3 hominoids, 8 Old World monkeys and 2 New World monkeys, for which the *NAT* homologous sequences were determined: *Pan paniscus* (bonobo), *Hylobates lar* (white-handed gibbon), *Nomascus gabriellae* (yellow-cheeked gibbon, *Macaca sylvanus* (barbary macaque), *Mandrillus sphinx* (mandrill), *Cercopithecus diana* (diana monkey), *Chlorocebus tantalus* (tantalus monkey), *Erythrocebus patas* (patas monkey), *Allenopithecus nigroviridis* (allen’s swamp monkey), *Colobus guereza* (guereza), *Presbytis cristatus* (silvered-leaf monkey), *Pithecia pithecia* (white-faced saki), and *Cebus apella* (tufted capuchin).

We designed amplification primers based on conserved sequences in the multiple alignments of database sequences retrieved from the genomes of *Homo sapiens*, *Pan troglodytes*, *Gorilla gorilla*, *Pongo pygmaeus*, *Macaca mulatta*, and *Papio hamadryas*. Separate alignments for sequences homologous to human *NAT1*, *NAT2* and *NATP* were as considered. Each newly obtained sequence was added to the alignments to refine primer specificity. The list of all primers used in the present study is provided in Additional file 8: Table S4. PCR reactions were carried out in 25 μl final volume, containing 25 ng of genomic DNA, 10 mM of each primer, 7.5 mM MgCl2, 2.5 mM dNTP mix, 1U GoTaq® DNA polymerase (Promega), and H_2_O. PCR conditions were as follows: denaturation at 95°C for 10 min, followed by 38 cycles at 94°C for 1 min (denaturation), 55°C for 50 s (annealing temperature-variable depending on the set of primers used, see Additional file 9: Table S5) and 72°C for 2 min (elongation), with a final 10 min elongation step at 72°C. Depending on the primate species, different primer pairs and annealing temperatures were used to optimize PCR conditions (Additional file 6: Table S2). PCR products were purified from agarose gels with the QIAquick Gel extraction kit and were sequenced in both directions by Millegen company (Labège-France) on an ABI 3730XL capillary sequencer (Applied Biosystems). In the case of non-specific amplifications, PCR products were cloned using the pGEM®-T Easy Vector System (Promega), according to manufacturer’s instructions, and both strands of the cloned fragments were sequenced using the M13F and M13R universal primers.

### Protein and codon sequence alignments

After discarding a few very divergent sequences that aligned poorly (*Anolis carolinensis NAT3*, *Oryzias latipes NAT3*, *Fugu rubripes NAT1*, *Tetraodon nigroviridis NAT1*), the remaining set of *NAT* ORF sequences were translated using the ExPASy Translate tool (http://web.expasy.org/translate/) and the amino acid sequences were aligned using ClustalW [61] and Multalin [62]. Both programs yielded similar results. The amino acid alignments were then converted to the corresponding alignments of codons using the PAL2NAL program [63]. These nucleotide alignments were used as inputs for the construction of phylogenetic trees, the analysis of gene conversion and tests of positive selection.

### Phylogenetic analysis

Phylogenetic trees were built from two nucleotide alignments: the first one included the 77 vertebrate *NAT* sequences retrieved from genomic databases (referred to as the ‘vertebrate dataset’) and the second one was composed of 58 primate sequences, including both the 17 database sequences and the 41 newly generated ones (referred to as the ‘primate dataset’). Phylogenetic relationships of *NAT* sequences were reconstructed using the maximum likelihood method implemented in PhyML v2.4.4 [64], after determining the optimal model of sequence substitution with Modeltest 3.04 [65]. For both datasets, a general time reversible model with a proportion of invariant sites and gamma distributed among-site rate variation (GTR + I + G) was used, as selected by the Akaike information criterion. The initial tree was determined by neighbor-joining (BIONJ). Branch supports were estimated from 1,000 PhyML bootstrap replicates using the same settings. Trees were displayed and colored with Bonsai 1.2 (http://depts.washington.edu/jtlab/software/softwareIndex.html).

### Detection of gene conversion

Possible recombination and gene conversion events were detected using GeneConv version 1.18 [66,67]. The program tests for gene conversion by finding identical fragments between pairs of sequences in a nucleotide alignment. Global and pairwise *P*-values are calculated in order to assess the statistical significance of the observed fragment lengths. Global *P*-values are more conservative because they are based on the comparison of each fragment with all possible fragments for the entire alignment. GeneConv was run using a group structure defining a distinct group for each set of *NAT* coding sequences within a species so as to look only for within-species gene conversion events and adjust Bonferroni corrections accordingly. All the default settings were used except for the mismatch penalty (set to 2) and the number of random permutations to compute global and pairwise *P*-values (set to 1,000,000). Only gene conversion events with global permutation *P*-values ≤ 0.01 were retained. In order to avoid confounding effects due to strong selection or recent mutation, GeneConv was also run using only silent sites.

Intragenic recombination events were also detected using five other methods implemented in the RDP3 v.3.44 software package [68]: two of these are phylogenetic methods, which infer recombination when different parts of the genome result in discordant topologies (RDP [69] and Bootscan [70]), while the other three are nucleotide substitution methods, which examine the sequences either for a significant clustering of substitutions or for a fit to an expected statistical distribution: MaxChi [71], Chimaera [72], and SiScan [73]. Common settings for all methods were to consider sequences as linear, to require phylogenetic evidence, to polish breakpoints and to check alignment consistency. RDP was run with default settings modified to use internal and external reference sequences and a window size of 30 bp. Default settings were used for MaxChi and Chimaera analyses. Bootscan default settings were modified to perform 1000 bootstrap replicates with cutoff percentage set to 95%. SiScan was run using a window size of 200 bp and a step size of 50 bp. *P*-values from individual algorithms were Bonferroni corrected to account for both multiple testing within each method and for multiple tests by different methods. Only events with corrected *P*-values < 0.01 were considered and the detected events were filtered to eliminate predictions of interspecies recombination.

### Maximum likelihood tests of positive selection

The ratio of nonsynonymous (amino acid altering) to synonymous (silent) substitution rates (dN/dS or ω) was used to evaluate the selective pressures acting on the *NAT* coding region, expecting dN/dS = 1 for neutrality, dN/dS < 1 for purifying selection, and dN/dS >1 for positive selection. The analysis was performed using the codeml tool of the PAML package version 4.2b [26,74], which allows pairs of nested models with and without positive selection to be tested in a likelihood ratio framework to determine if adaptive evolution has occurred. We used two pairs of site-evolution models to test whether some sites (codons) are under positive selection: (1) the M1a (Nearly Neutral) and M2a (Positive Selection) models and (2) the M7 (beta) and M8 models (beta & ω). M1a allows sites to fall into two categories with ω < 1 (purifying selection) and ω = 1 (neutral evolution), whereas model M2a extends model M1a with a further class with ω > 1 (positive selection) [75,76]. M7 allows ten classes of ω sites between 0 and 1 according to a beta distribution with parameters *p* and *q*, whereas M8 adds an additional class with ω possibly > 1 as M2a does. Candidate sites for positive selection were pinpointed using the BEB approach described by Yang et al. [77], which calculates the posterior probability that each codon site falls into a site class affected by positive selection (in models M2a and M8) and provides the posterior ω values which represent the strength of natural selection acting on each residue. The heterogeneity of the dN/dS ratio among lineages (branch-based test) was tested by comparing the free-ratio model, which assumes as many ω parameters as the number of branches in the tree, to the one-ratio null model M0 which assumes only one ω value for all branches [78]. Finally, we used the branch-site test of selection developed by Zhang et al. [79] to detect episodic positive selection along prespecified lineages on a phylogeny that affects only a few sites in the protein-coding gene. By focusing on individual amino acid residues and particular lineages, this test should provide greater power than site-based tests, which average substitution rates over all branches on the phylogeny, or branch-based tests, which average over all codons in the gene [79-81]. In this test, the branch being tested for positive selection is called the foreground branch, and the other branches in the tree are called the background branches. In the null model, all codons in the foreground and background branches are assumed to evolve under negative selection (ω < 1) or neutrally (ω = 1), whereas in the alternative model A, the foreground branch also includes sites evolving under positive selection (ω > 1). As described above, the posterior probability that each site falls into the positively selected class was calculated using the BEB method. Branch-site tests were also carried out at the clade level by performing a multiple branch site analysis where all the branches within a clade are grouped as the foreground lineage and the remaining branches in the tree are grouped as background [82]. This approach allows testing adaptive evolution in multiple branches while avoiding the assumptions of the clades analysis model (CmC, [83]) which has been shown to have a high false-positive rate and to be highly unreliable when faced with moderate among-site ω variation [84].

In all PAML analyses, model comparisons were made using LRTs. For each of the LRTs, twice the log-likelihood difference between alternative and null model (2Δln*L*) was compared to critical values from a χ^2^ distribution with degrees of freedom equal to the difference in the number of estimated parameters between both models [75]. In branch-site tests, we used the conservative $X_{1}^{2}$ distribution to conduct the LRT and the Bonferroni correction was employed to control the type I error rate when comparing multiple foreground lineages. To avoid local optima and ensure that the global peak is found, each model was run three times with different ω starting values. The three runs always produced identical results. To identify codon sites under positive selection along specific branches of particular interest, we inferred ancestral sequences using baseml (model = HKY85, Mgene = 4; cleandata = 0) and identified nonsynonymous changes by pairwise comparison of sequences. In all PAML analyses, codons containing alignment gaps due to insertion-deletion events among species were treated as missing data and not considered in pairwise comparisons where one species contained sequence and another species did not. We used the F3 × 4 codon model of Goldman and Yang [85] which is very similar to the nucleotide model HKY85 with different substitution rates and base frequencies for the three codon positions. The analyses were applied to several different partitions of the data, as described in the Results section.

## Abbreviations

BEB: Bayes empirical Bayes; CoA: Coenzyme A; LRT: Likelihood ratio test; ML: Maximum-likelihood; NAT: Arylamine N-acetyltransferase; ORF: Open reading frame; pABA: *p*-aminobenzoic acid; pABG: *p*-aminobenzoylglutamate; pASA: *p*-aminosalicylic acid; PP: Posterior probabilities.

## Competing interests

The authors declare that they have no competing interests.

## Authors’ contributions

AS carried out the evolutionary and statistical analyses performed in the study and drafted the manuscript. JM, CV and EL carried out the molecular genetic analyses. SB and EP participated in the study design and coordination and helped to draft the manuscript. PD and BC conceived of the study, participated in its design and coordination and helped to draft the manuscript by revising it critically. All authors read and approved the final manuscript.

## Supplementary Material

Additional file 1 **Alignment result for the set of 77 vertebrate *NAT *nucleotide sequences.** Click here for file

Additional file 2: Figure S1 Maximum likelihood tree of vertebrate *NAT*s obtained using PhyML after removing all regions affected by gene conversion from the multiple nucleotide alignment (converted fragments were replaced by ‘?’ characters in the alignment). A general time reversible model with a proportion of invariant sites and gamma distributed among-site rate variation (GTR + I + G) was used, as selected by Akaike information criterion in Modeltest 3.04. The tree is rooted with the three fish species (*Danio rerio*, *Gasterosteus aculeatus*, *Oryzias latipes*) and bootstrap values of 1,000 replicates are shown as percentages at nodes. Bootstrap values are only shown for nodes with greater than 50% support. The clades of Afrotheria, Laurasiatheria, Lagomorpha, Rodentia and Primates are shown in aqua blue, purple, green, blue and red, respectively. Click here for file

Additional file 3: Figure S2 Gene order and orientation in regions surrounding the *NAT* genes on human (*Homo sapiens*) and mouse (*Mus musculus*) chromosome 8. Gene lengths and intergenic distances are not drawn to scale. Double slashes (//) indicate continuing sequence data extending toward the centromeric (cen) and telomeric (tel) parts of the chromosome. Grey boxes indicate *NAT*-like sequences. The region encompassing the three *NAT* loci spans approximately 190 kb in human, but less than 60 kb in mouse. In humans, two of the loci are functional (*NAT1* and *NAT2*) and one is a pseudogene (*NATP*). In mice, all three appear to generate functional transcripts, although no apparent specific substrate for the product of the third locus (*Nat3*) has been identified. Interestingly, Mouse *Nat2* is considered as the functional equivalent of human *NAT1*, based on substrate profile, tissue distribution and expression during development (see [10] for review). Click here for file

Additional file 4: Table S1 Patterns of nucleotide substitutions along the four branches showing evidence of positive selection in the *Pteropus vampyrus* clade of *NAT* genes. Click here for file

Additional file 5 **Alignment result for the set of 58 primate *NAT *nucleotide sequences.** Click here for file

Additional file 6: Table S2 Posterior mean of ω as estimated by the codeml program for different regions and different categories of sites in the NAT protein for the four datasets investigated. Click here for file

Additional file 7: Table S3 Comparisons of the mean ω between different regions and categories of sites within the NAT protein sequence. Click here for file

Additional file 8: Table S4 Primers for amplification and sequencing of primate *NAT* genes. Click here for file

Additional file 9: Table S5 Primer pairs and annealing temperatures used for each primate species for the PCR amplification of the three *NAT* genes. Click here for file
