# Supplementary figures and images for: Rapid birth-and-death evolution of the xenobiotic metabolizing NAT gene family in vertebrates with evidence of adaptive selection
Source: BMC Evol Biol. 2013 Mar 7;13:62. doi: 10.1186/1471-2148-13-62 (PMC3601968; doi:10.1186/1471-2148-13-62)

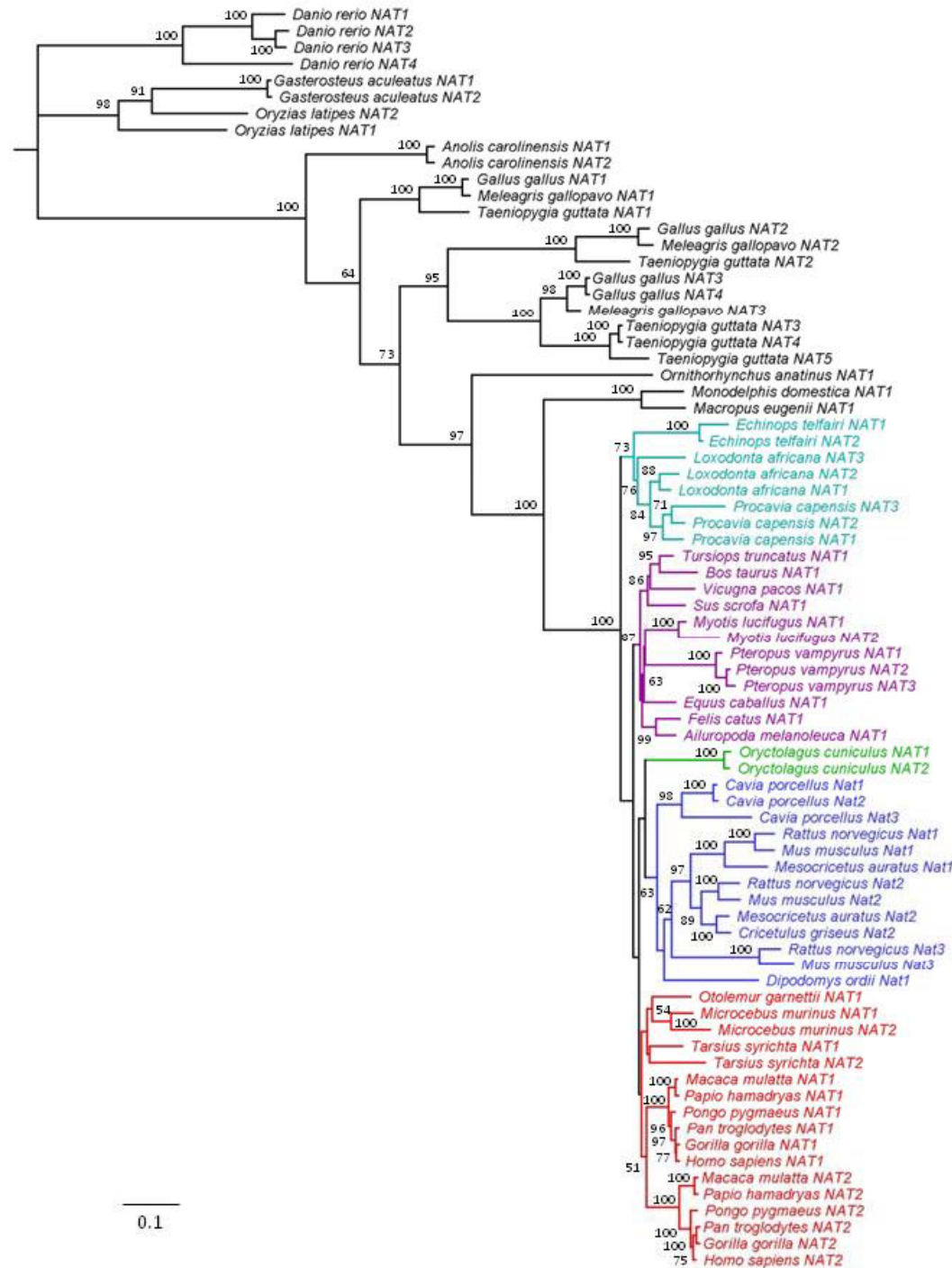

Afrotheria

Laurasiatheria

Lagomorpha

Rodentia

Primates

Figure S1

0.1

Supplement: Additional file 2: Figure S1 — Maximum likelihood tree of vertebrate NATs obtained using PhyML after removing all regions affected by gene conversion from the multiple nucleotide alignment (converted fragments were replaced by ‘?’ characters in the alignment). A general time reversible model with a proportion of invariant sites and gamma distributed among-site rate variation (GTR + I + G) was used, as selected by Akaike information criterion in Modeltest 3.04. The tree is rooted with the three fish species (Danio rerio, Gasterosteus aculeatus, Oryzias latipes) and bootstrap values of 1,000 replicates are shown as percentages at nodes. Bootstrap values are only shown for nodes with greater than 50% support. The clades of Afrotheria, Laurasiatheria, Lagomorpha, Rodentia and Primates are shown in aqua blue, purple, green, blue and red, respectively. [file 1471-2148-13-62-S2.pdf]

*H. sapiens*  
Chr 8

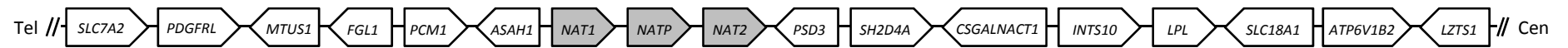

*M. musculus*  
Chr 8

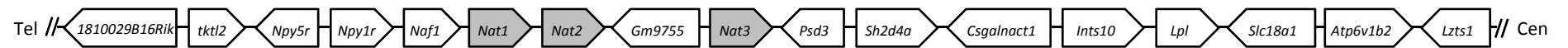

Figure S2

Supplement: Additional file 3: Figure S2 — Gene order and orientation in regions surrounding the NAT genes on human (Homo sapiens) and mouse (Mus musculus) chromosome 8. Gene lengths and intergenic distances are not drawn to scale. Double slashes (//) indicate continuing sequence data extending toward the centromeric (cen) and telomeric (tel) parts of the chromosome. Grey boxes indicate NAT-like sequences. The region encompassing the three NAT loci spans approximately 190 kb in human, but less than 60 kb in mouse. In humans, two of the loci are functional (NAT1 and NAT2) and one is a pseudogene (NATP). In mice, all three appear to generate functional transcripts, although no apparent specific substrate for the product of the third locus (Nat3) has been identified. Interestingly, Mouse Nat2 is considered as the functional equivalent of human NAT1, based on substrate profile, tissue distribution and expression during development (see [10] for review). [file 1471-2148-13-62-S3.pdf]
