# Supplementary material for: Rapid birth-and-death evolution of the xenobiotic metabolizing NAT gene family in vertebrates with evidence of adaptive selection
Source: BMC Evol Biol. 2013 Mar 7;13:62. doi: 10.1186/1471-2148-13-62 (PMC3601968; doi:10.1186/1471-2148-13-62)
Supplement: Additional file 4: Table S1 — Patterns of nucleotide substitutions along the four branches showing evidence of positive selection in the Pteropus vampyrus clade of NAT genes. [file 1471-2148-13-62-S4.doc]

**Table S1 Patterns of nucleotide substitutions along the four branches showing evidence of positive selection in the *Pteropus vampyrus NAT*s clade**

| Branch^a^ | ω^b^ | dN/dS^b^ | Nucleotide changes along the branch^c^ | List of nonsynonymous changes^c^ |
| --- | --- | --- | --- | --- |
|  |  |  |  |  |
| i | 2.85 | 10.7/1.4 | 11 nucleotide changes of which 10 are nonsynonymous and affect 9 distinct codon sites. | L67V, H73Q, I93M, A123V, D179V, L180F, T211M, P228L, R286G. |
| j | 2.54 | 14.0/2.0 | 16 nucleotide changes of which 14 are nonsynonymous and affect 13 distinct codon sites. | L69I, T96I, P97Q, S128F, M131I, L209Y, Q210L, K213E, A214T, T218K, N219K, K220N, V235L. |
| k | ∞ | 4.9/0.0 | 5 nucleotide changes, all nonsynonymous. | M48L, I50F, L108P, L267M, R286Q. |
| l | 2.43 | 13.4/2.0 | 15 nucleotide changes of which 13 are nonsynonymous and affect 12 distinct codon sites. | R33Q, L37F, T55F, L180I, F204I, L206A, T211R, V216I, C233Y, H283Q, D284G, D285G. |
|  |  |  |  |  |

^a^ Branches are labeled as shown in Figure 3.

^b^ ω ratio and numbers of nonsynonymous to synonymous changes as estimated by the free-ratio model in the codeml program of the PAML package.

^c^ Deduced from the pairwise comparison of sequences at the basal and peripheral ends of each branch (ancestral sequences at nodes were inferred using the baseml program of the PAML package). Note that two non-silent nucleotides changes affecting the same codon can yield to a single nonsynonymous change at a single codon site.
