# Supplementary material for: Rapid birth-and-death evolution of the xenobiotic metabolizing NAT gene family in vertebrates with evidence of adaptive selection
Source: BMC Evol Biol. 2013 Mar 7;13:62. doi: 10.1186/1471-2148-13-62 (PMC3601968; doi:10.1186/1471-2148-13-62)
Supplement: Additional file 6: Table S2 — Posterior mean of ω as estimated by the codeml program for different regions and different categories of sites in the NAT protein for the four datasets investigated. [file 1471-2148-13-62-S6.doc]

**Table S2** **Posterior mean of ω as estimated by the codeml program for different regions and different categories of sites in the NAT protein for the four datasets investigated**

|  | **Vertebrates** | **Mammals** | **Primates** | **Simian NAT2** |
| --- | --- | --- | --- | --- |
|  |  |  |  |  |
| Full NAT sequence (*n* = 290) | 0.34 ± 0.30 | 0.40 ± 0.35 | 0.47 ± 0.35 | 0.66 ± 0.61 |
| Domain I (*n* = 83) | 0.25 ± 0.23 | 0.31 ± 0.27 | 0.34 ± 0.29 | 0.51 ± 0.30 |
| Domain II (*n* = 109) | 0.36 ± 0.32 | 0.42 ± 0.36 | 0.52 ± 0.38 | 0.77 ± 0.79 |
| Interdomain (*n* = 37) | 0.48 ± 0.39 | 0.55 ± 0.45 | 0.56 ± 0.35 | 0.62 ± 0.46 |
| Domain III (*n* = 61) | 0.36 ± 0.29 | 0.44 ± 0.34 | 0.51 ± 0.34 | 0.68 ± 0.60 |
| 17-residue insert (*n* = 17) | 0.44 ± 0.24 | 0.48 ± 0.27 | 0.58 ± 0.34 | 0.77 ± 0.87 |
| C-terminal tail (*n* = 15) | 0.53 ± 0.37 | 0.53 ± 0.37 | 0.68 ± 0.23 | 0.90 ± 0.58 |
| CoA binding sites (*n* = 16) | 0.70 ± 0.50 | 0.77 ± 0.52 | 0.73 ± 0.42 | 0.95 ± 0.84 |
| Substrate binding sites (*n* = 11) | 0.41 ± 0.41 | 0.47 ± 0.44 | 0.69 ± 0.47 | 1.31 ± 1.11 |
|  |  |  |  |  |

Values are expressed as mean ± standard deviation. *n*, number of sites.
