# Supplementary material for: Rapid birth-and-death evolution of the xenobiotic metabolizing NAT gene family in vertebrates with evidence of adaptive selection
Source: BMC Evol Biol. 2013 Mar 7;13:62. doi: 10.1186/1471-2148-13-62 (PMC3601968; doi:10.1186/1471-2148-13-62)
Supplement: Additional file 7: Table S3 — Comparisons of the mean ω between different regions and categories of sites within the NAT protein sequence. [file 1471-2148-13-62-S7.doc]

**Table S3** **Comparisons of the mean ω between different regions and categories of sites within the NAT protein sequence**

|  | **Vertebrates** | **Mammals** | **Primates** | **Simian NAT2** |
| --- | --- | --- | --- | --- |
|  |  |  |  |  |
| Heterogeneity between protein domains (I, II, III, Interdomain) | 0.002** | 0.03* | 0.0008*** | 0.03* |
| Domain I vs. Domain II | 0.006** | 0.03* | 0.0003*** | 0.002** |
| Domain I vs. Domain III | 0.004** | 0.02* | 0.003** | 0.04* |
| Domain I vs. the rest of the sequence | 0.0005*** | 0.006** | < 0.0001*** | 0.005** |
| Domain II vs. the rest of the sequence | 0.30 | 0.53 | 0.05 | 0.05 |
| Domain III vs. the rest of the sequence | 0.24 | 0.21 | 0.25 | 0.67 |
| Interdomain vs. the rest of the sequence | 0.03* | 0.11 | 0.11 | 0.98 |
| 17-residue insert vs. the rest of the sequence | 0.02* | 0.06 | 0.18 | 0.56 |
| C-terminal tail vs. the rest of the sequence | 0.007** | 0.06 | 0.01* | 0.03* |
| CoA binding sites vs. the remaining sites of the sequence | 0.002** | 0.003** | 0.01* | 0.18 |
| Substrate binding sites vs. the remaining sites of the sequence | 0.97 | 0.87 | 0.19 | 0.04* |
|  |  |  |  |  |

The heterogeneity of ω between the four protein domains is tested by a Kruskal-Wallis analysis of variance, and the pairwise comparisons by a Mann-Whitney test. **P* < 0.05; ***P* < 0.01; ****P* < 0.001.
