# Supplementary material for: Rapid birth-and-death evolution of the xenobiotic metabolizing NAT gene family in vertebrates with evidence of adaptive selection
Source: BMC Evol Biol. 2013 Mar 7;13:62. doi: 10.1186/1471-2148-13-62 (PMC3601968; doi:10.1186/1471-2148-13-62)
Supplement: Additional file 8: Table S4 — Primers for amplification and sequencing of primate NAT genes. [file 1471-2148-13-62-S8.doc]

**Table S4** **Primers for amplification and sequencing of primate *NAT* genes**

|  | Name of primer | Sequence | Sense |
| --- | --- | --- | --- |
| *NAT1* | Nat1-A | 5' AGCCATAATTAGCCTACTC 3' | Forward |
|  | Nat1-A2 | 5' ATCATGGACATTGAAGCATA 3' | Forward |
|  | Nat1-AR | 5' GTACAGAAGATACATGATAGG 3' | Reverse |
|  | Nat1-A2R | 5' CACCATGTTTGGGCACAAGC 3' | Reverse |
| *NAT2* | Nat2-A | 5' GACAGATACTTATAACCATTG 3' | Forward |
|  | Nat2-AR | 5' GTTGGGTGATACATACACAAG 3' | Reverse |
| *NATP* | Np-L2 | 5' GAATCTAAGGGCAAAAGTAATG 3' | Forward |
|  | Np-L3 | 5' GAGAAATTCATGCAATAAAAGT 3' | Forward |
|  | Np-L4 | 5' ACGGCAAGTACCACATCACT 3' | Forward |
|  | Np-L5 | 5' GGGATCAWGGACATTAYAGT 3' | Forward |
|  | Np-L6 | 5' ATCTTCAGCAACAGGTCTGA 3' | Forward |
|  | Np-R1 | 5' AATCTTCAATTGTTCAGGATTC 3' | Reverse |
|  | Np-R2 | 5' TGAAAATGTGTGGTTATCATTC 3' | Reverse |
