# Supplementary material for: Rapid birth-and-death evolution of the xenobiotic metabolizing NAT gene family in vertebrates with evidence of adaptive selection
Source: BMC Evol Biol. 2013 Mar 7;13:62. doi: 10.1186/1471-2148-13-62 (PMC3601968; doi:10.1186/1471-2148-13-62)
Supplement: Additional file 9: Table S5 — Primer pairs and annealing temperatures used for each primate species for the PCR amplification of the three NAT genes. [file 1471-2148-13-62-S9.doc]

**Table S5 Primer pairs and annealing temperatures used for each primate species for the PCR amplification of the three *NAT* genes**

| **Species** | ***NAT1*** | ***NAT2*** | ***NATP*** |
| --- | --- | --- | --- |
|  |  |  |  |
| *Cebus apella* | Nat1-A2, Nat1-A2R 48°C | Nat2-A, Nat2-AR 51°C | - |
| *Pithecia pithecia* | Nat1-A, Nat1-AR 50°C | Nat2-A, Nat2-AR 48°C | - |
| *Colobus guereza* | Nat1-A, Nat1-AR 50°C | Nat2-A, Nat2-AR 50°C | Np-L3, Np-R1 50°C Np-L5, Np-R2 55°C |
| *Presbytis cristatus* | Nat1-A, Nat1-AR 55°C | Nat2-A, Nat2-AR 50°C | Np-L3, Np-R1 50°C Np-L5, Np-R2 55°C |
| *Macaca sylvanus* | Nat1-A, Nat1-AR 53°C | Nat2-A, Nat2-AR 53°C | Np-L3, Np-R1 50°C Np-L4, Np-R2 58°C |
| *Mandrillus sphinx* | Nat1-A, Nat1-A2R 51°C | Nat2-A, Nat2-AR 53°C | Np-L3, Np-R1 50°C Np-L4, Np-R2 56°C |
| *Allenopithecus nigroviridis* | Nat1-A, Nat1-AR 54°C | Nat2-A, Nat2-AR 51°C | Np-L3, Np-R1 50°C Np-L6, Np-R2 56°C |
| *Cercopithecus diana* | Nat1-A, Nat1-AR 55°C | Nat2-A, Nat2-AR 55°C | Np-L3, Np-R1 50°C Np-L6, Np-R2 53°C |
| *Chlorocebus tantalus* | Nat1-A, Nat1-AR 55°C | Nat2-A, Nat2-AR 55°C | Np-L3, Np-R1 50°C Np-L6, Np-R2 56°C |
| *Erythocebus patas* | Nat1-A, Nat1-AR 55°C | Nat2-A, Nat2-AR 55°C | Np-L3, Np-R1 50°C Np-L6, Np-R2 56°C |
| *Hylobates lar* | Nat1-A, Nat1-A2R 51°C | Nat2-A, Nat2-AR 55°C | - |
| *Nomascus gabriella* | Nat1-A, Nat1-A2R 51°C | Nat2-A, Nat2-AR 55°C | - |
| *Pan paniscus* | Nat1-A, Nat1-AR 53°C | Nat2-A, Nat2-AR 55°C | Np-L2, Np-R2 55°C |
|  |  |  |  |
